# Analyzing sentiments and themes on cannabis in Canada using 2018 to 2020 Twitter data

**DOI:** 10.1186/s42238-022-00132-1

**Published:** 2022-04-13

**Authors:** Maisam Najafizada, Arifur Rahman, Jennifer Donnan, Zhihao Dong, Lisa Bishop

**Affiliations:** 1 Faculty of Medicine, Memorial University, 300 Prince Phillip Drive, St. John’s, Newfoundland and Labrador A1B 3 V6 Canada; 2 School of Pharmacy, Memorial University, St. John’s, Newfoundland and Labrador Canada; 3 Department of Computer Science, Memorial University, St. John’s, Newfoundland and Labrador Canada

**Keywords:** Cannabis, Twitter, Cannabis Policy, Social media, Sentiment analysis, Content analysis

## Abstract

**Introduction:**

The Canadian Cannabis Act came into effect on October 17, 2018, which allowed Canadian adults to consume cannabis for non-medical purposes (Government of Canada, Cannabis regulations (SOR/2018-144). Cannabis Act, (2018a); Parliament of Canada, C-45: an Act respecting cannabis and to amend the Controlled Drugs and Substances Act, the Criminal Code and other Acts, 2018). With this major policy change, it is unknown how the attitude of the public changed and how information on cannabis changed. Social media platforms, including Twitter, are significant venues for studying emerging patterns in social issues such as cannabis legalization. This study aimed to examine sentiments, themes and contents of cannabis-related tweets by suppliers (both licensed and unlicensed) and general tweets in Canada. To our knowledge, this is the first analysis that mixes sentiment analysis and thematic analysis of Canadians’ cannabis-related Twitter data.

**Method:**

A sample of Canadian cannabis-related tweets was collected from January 2018 to August 2020 through the Twitter Application Programming Interface (API). Using a standard access token and the Twitter Standard Search API, tweets were extracted based on Twitter handles to capture the content of both licensed and unlicensed cannabis retailers in Canada, as well as relevant cannabis-related keywords to capture public content. We conducted sentiment and positive polarity analyses, and content analysis to identify attitudes and themes around cannabis use in Canada.

**Results:**

This study gathered and analyzed a total of 44,970 tweets in the sentiment analysis and a total of 1035 tweets in the thematic analysis. Descriptive analysis showed that monthly tweets peaked prior to legalization in October 2018 and again during the initial wave of the COVID-19 pandemic in February and March 2020. The data showed an overall positive sentiment polarity with a high of + 0.24 in April 2019 and a low of + 0.14 in March 2020.

Thematic analysis revealed the themes: (i) education/information, (ii) uses of cannabis, (iii) cannabis products including packing, quality, price, types, and sources, (iv) cannabis policies including regulations and public safety, (v) access, (vi) social issues include gender and stigma, and (vii) COVID-19 impact.

**Conclusion:**

This study combined the power of big data collection and analysis with manual coding and analysis methods to extract rich content from large data using social media communications on issues related to cannabis in Canada. The findings of this study may inform policies on advertising cannabis products and highlighted some patterns related to education, access, and safety that deserve further investigation.

## Introduction

The Canadian Cannabis Act came into effect on October 17, 2018, which allowed Canadians 19 years of age or older (in Alberta 18+ and in Quebec 21+) to consume cannabis for non-medical purposes (Government of Canada 2018a; Parliament of Canada 2018). The purpose of the Cannabis Act is to ensure safe access to cannabis for Canadians and to protect their health and safety (Government of Canada 2018a, b, c). However, data from the National Cannabis Survey suggests that unregulated cannabis sales are still prevalent even though licensed retailers are supplying their products (Canadian Centre on Substance Abuse and Addiction 2020). Cannabis is widely used among all age groups, especially youths and young adults. According to the National Cannabis Survey, consumption of non-medical cannabis has increased among people aged 15 to 24 years consistently over the last 6 years (Canadian Centre on Substance Abuse and Addiction 2020; Government of Canada 2018b). Before legalization, 46.6% of Canadians reported using cannabis at least once in their lifetime (Government of Canada 2018c). The Canadian Tobacco, Alcohol, and Drugs (CTADS) Survey in 2017 reported that 14.8% population used cannabis at least once in the previous year, which increased to 16.7% after legalization (Government of Canada 2020). A Canadian Centre on Substance Use and Addiction (CCSA) study in 2022 reported that the consumption method was shifting from smoking to edible products, and purchases from licensed retailers have been increasing since the two most common avenues of purchase in 2020 were legal physical and online stores (Canadian Centre on Substance Abuse and Addiction 2022).

Social media platforms are significant venues for studying emerging patterns in social issues such as cannabis legalization. The messages generated by users, producers, and suppliers have the potential to reflect trends in real-time (Darren et al. 2020; Moreno et al. 2018; Jenkins et al. 2021). The social media accounts of cannabis retailers have been studied on multiple platforms including Facebook (Moreno et al. 2018; Jenkins et al. 2021), Instagram (Jenkins et al. 2021), and Twitter (Cavazos-Rehg et al. 2014, Daniulaityte et al. 2016; Lamy et al. 2016). Twitter, an extensively used social networking platform, offers an excellent opportunity to investigate public sentiment and perceptions on social issues including cannabis use (Darren et al. 2020). Previous studies using tweets addressed cannabis use among youth, minorities (Cabrera-Nguyen et al. 2016; Cavazos-Rehg et al. 2014), and geographic variations (Daniulaityte et al. 2015). Prior research also characterized the users’ sentiment from cannabis-related tweets (Cavazos-Rehg et al. 2014, Daniulaityte et al. 2016; Lamy et al. 2016). The licensed and unlicensed cannabis suppliers in Canada use tweets to attract and communicate with consumers. Therefore, analysis of cannabis-related tweets from both suppliers and consumers are important avenues to understand their views and attitudes towards cannabis consumption.

Previous studies used several approaches to analyze social media users’ sentiments from the unstructured text data (Hutto and Gilbert  2014; Liu and Young 2018). Sentiment analysis is a useful tool to determine public attitudes toward a social issue. Though sentiment analysis can present the users’ positive, negative, or neutral attitudes, it does not include anything about the content of the data. To explore the content of the text and the details of public perception, thematic content analysis can provide greater insight (O’Callaghan et al. 2015).

Previous Twitter content analysis of cannabis revealed themes such as users’ intent to use, health benefits, sex/romance, and legalization (Cavazos-Rehg et al. 2015). Moreno et al. (2018) evaluated the social media content (Facebook and Twitter) of six cannabis companies to assess whether the messages complied with the Washington Administrative Code on cannabis and found some violations of regulations on messaging. A study of Twitter content about cannabis and alcohol that resulted in a high number of re-tweets found that the use and effects of cannabis were the most common themes (West et al. 2012). However, most of these previous studies were focused on tweets from adolescents and influential Twitter users (Cabrera-Nguyen et al. 2016; Cavazos-Rehg et al. 2014; Cavazos-Rehg et al. 2015; Krauss et al. 2016). Another study used tweets from a sample of the general population, which captured topics such as cannabis use, packaging, health effects, the cannabis industry, and legalization (Allem et al. 2020). As more jurisdictions legalize non-medical cannabis, the cannabis-related policy landscape continues to rapidly change, so do the attitudes and perceptions toward cannabis.

This study aimed to examine the themes and contents of cannabis-related tweets by suppliers (both licensed and unlicensed) and cannabis users in Canada. To our knowledge, this is the first thematic analysis of Canadians’ cannabis-related Twitter data.

## Method

A sample of cannabis-related tweets was collected from January 2018 to August 2020 through the Twitter Application Programming Interface (API). This period enabled us to capture data before and after legalization. Twitter handles of licensed and unlicensed retailers were identified manually. Two summer students (Acknowledged below) searched provincial government websites to identify licensed retailers, and then hand-searched Twitter to locate their Twitter handles. For unlicensed retailers, an informal list of Canadian unlicensed retailers was located online and then each retailer’s name was hand-searched to find their Twitter handles. The final list of 30 (19 licensed and 11 unlicensed) retailers was made based on the number of their followers (Appendix 1). Then, using a standard access token and the Twitter Standard Search API, tweets relevant to this study were extracted based on Twitter handles to capture the content of both licensed and unlicensed cannabis retailers in Canada as shown in Appendix 1, and 80 keywords (e.g., “Cannabis,” “Ganja,” “Weed,” “marijuana,” “Indica”) as shown in Appendix 2, to capture user content. For every tweet, we gathered a list of fields including user ID, tweet content, user Twitter handle, number of times it was favorited, retweet count, and others as shown in Appendix 3.

Once the corpus of tweets was gathered (44,970 tweets), we conducted sentiment and positive polarity analyses on the entire corpus of tweets using TextBlob: Simplified Text Processing Python library. TextBlob is a Python library for processing textual data, which provides a consistent API for common natural language processing tasks such as tokenization and sentiment analysis. Sentiment analysis in this paper quantifies the emotional content by assigning polarity scores to words or phrases. The polarity score ranges between − 1 and 1. Positive polarity scores indicate positive emotions, while negative polarity scores indicate negative emotions. The positive emotion rate within the range between 0 and 1, defined by the percentage of tweets with positive emotion, was another metric that we used to measure users’ overall sentiment.

The corpus of tweets was separated by their source, which included: licensed retailers’ Twitter accounts, unlicensed retailers’ Twitter accounts, and tweets gathered using keywords. These three groups of tweets were then used for further content analysis using Excel.

From the corpus of 44,970 tweets, a subset (1035 tweets) was extracted to conduct thematic content analysis. A content analysis was performed in several sequential steps. After data cleaning, the three groups of tweets by source categories (licensed, unlicensed, and general tweets using keywords) were ranked (highest to lowest) based on two categories: “most favorited” and “most retweeted.” The top 250 tweets most favorited and most retweeted in each category (500 each) were considered for manual coding. In total, 1500 most-favorited and most-retweeted tweets were compiled in an Excel Sheet for manual coding. All tweets by licensed retailers were posted after legalization and as such, it was used as a point of comparison.

Tweets were manually examined to identify nodes and themes. First, tweets “not related to cannabis” or “not in the context of Canada” were discarded. Any tweet that was not directly or indirectly about cannabis or its products (e.g., about COVID-19, or congratulatory messages) and any tweets that were explicitly about jurisdictions outside of Canada (e.g., USA or Europe) were removed from the list. Second, a large number of most-favorited and most retweeted tweets by unlicensed retailers were messages on “free giveaways.” Those tweets were tabled and reported separately. Since a large number of tweets fell under those categories, we decided to include additional 100 Tweets from each category to increase the corpus of data for manual coding as shown in Table 1. Subsequently, a thematic analysis was conducted on a total of 1035 tweets.

**Table 1:** Sampling tweets for content analysis

	Most-favorited			Most-retweeted			Grand total
	Number of tweets included in two rounds	Deleted irrelevant and repetitive tweets	Total	Number of tweets included in two rounds	Deleted irrelevant and repetitive tweets	Total	
**Cannabis licensed retailers**	250 + 100	154	**196**	250 + 100	217	**133**	**329**
**Cannabis unlicensed retailers**	250 + 100	150	**200**	250 + 100	174	**176**	**376**
**Keyword search**	250 + 100	183	**167**	250 + 100	187	**163**	**330**
		Total	**563**			**472**	**1035**

Thematic analysis was used to identify themes or patterns within the data to make sense of the contents (Braun and Clarke 2006). The unit of analysis was the whole tweet. In many cases, one tweet was coded under multiple themes. First, familiarization with the contents of the tweets generated ideas for creating codes. Each researcher (AR & MN) independently identified codes and met several times throughout the coding process to discuss the identified codes. After an agreement was reached on the final list codes, intercoder reliability was measured at 93%. Subsequently, those codes were grouped and linked with one another into themes and then shared with the larger team for discussion. Discrepancies were first discussed between AR & MN; any unresolved discrepancies were discussed with the larger team for resolution. Each theme was reviewed by two researchers (AR & MN) to ensure it reflected the associated codes and content of the tweets. Interpretations of the themes were discussed with the other researchers throughout the process to minimize the risk of preconceptions and subjectivity. Finally, the focus area and key insights of each tweet were discussed to present an overview of the findings. The study did not require ethics approval because the data was publicly available.

## Results

**Fig. 1:**
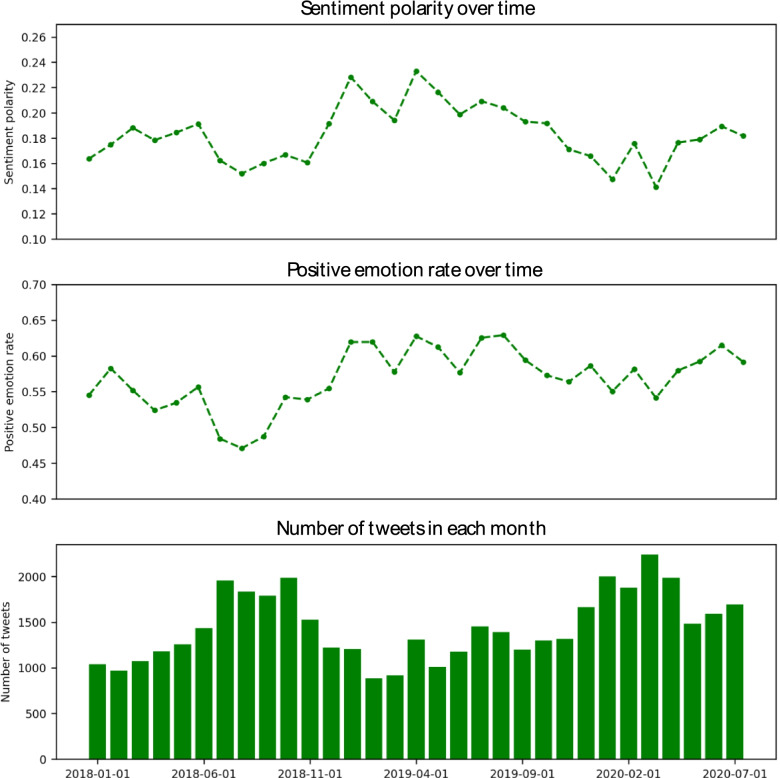
Sentiment polarity over time, positive emotions rate over time, and monthly tweets

This study gathered and analyzed a total of 44,970 tweets in the sentiment analysis. An initial descriptive analysis showed that monthly tweets peaked prior to legalization in October 2018 and again during the initial wave of the COVID-19 pandemic in February and March 2020 (Fig. 3). The data showed an overall positive sentiment polarity with a high of + 0.24 in April 2019 and a low of + 0.14 in March 2020. The positive emotion rate was lowest (+ 0.47) in Aug 2018 and highest (+ 0.64) in Aug 2019 (Fig. 1)

**Fig. 2:**
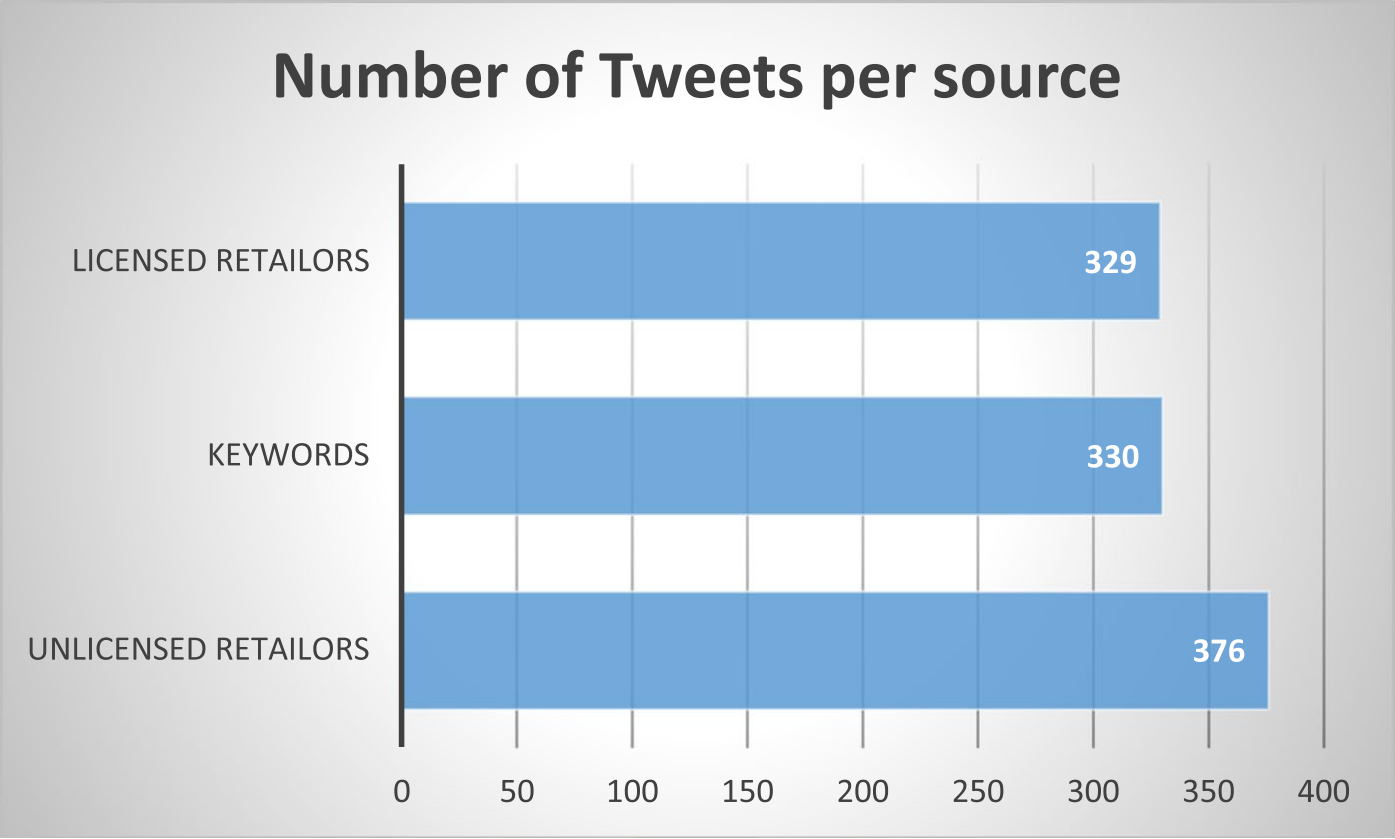
Distribution of tweets selected for analysis

The thematic content analysis was conducted on 1035 tweets from three sources (Fig. 2): (i) tweets gathered from licensed cannabis retailers, (ii) tweets gather from unlicensed cannabis retailers, and (iii) general tweets gathered through keyword search. The most favorited and most re-tweeted tweets were put together for each source.

**Fig. 3:**
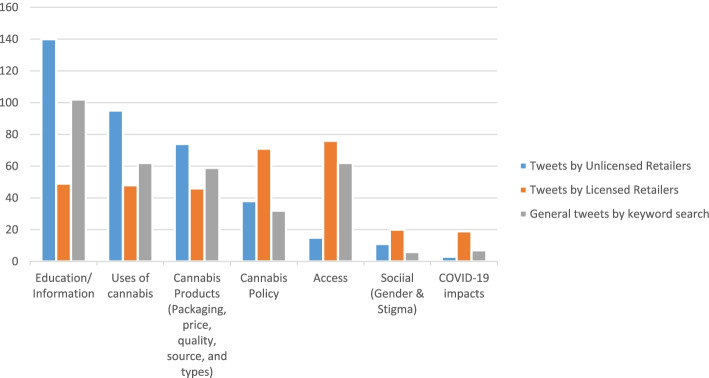
Thematic representation of the findings

The tweets from all three sources fell under the following themes (Fig. 3): (i) education/information, (ii) uses of cannabis, (iii) cannabis products including packing, quality, price, types, and sources, and (iv) cannabis policies including regulations and public safety, (v) access, (vi) social issues include gender and stigma, and (vii) COVID-19 impact.

An important pattern was that the licensed retailers (as a proxy for post-legalization tweets) mentioned access and policy more than others. Unlicensed retailers (as a proxy for pre-legalization tweets), focused more on education, uses of cannabis, and cannabis products, while they focused least on access and policy (Fig. 3). Social issues such as gender and stigma, and the impact of COVID-19 were other issues discussed. Of the seven themes shown in Fig. 3, we reported the most frequent themes by each source. Since all tweets by licensed retailers were after legalization, reporting tweet sources indicated some change in messaging before and after legalization.

### Cannabis licensed retailers

Approximately three quarters (75%) of the tweets from the licensed cannabis retailers could be described by four broad themes: access (23%), cannabis policy (22%), education/information (15%), and uses of cannabis (15%). The product-relevant tweets made up 14% of the data; among them, there was 5% on quality, 4% on source, and 3% on types of products. Six percent (6%) of the tweets from licensed cannabis retailers were related to COVID-19 and cannabis, which might be related to all other themes. About 6% of the tweets were related to stigma and gender. Table 2 shows the nodes in each theme. This report primarily focused on the four primary themes.

**Table 2:** Themes and nodes in licensed cannabis retailer

Themes	Nodes	Number and percentage of tweets
Access	Continuous access; easy access to store; limited access; online access; access to different formulations; developing mobile apps; barriers to online access; delay in delivery; home delivery; free delivery; increased hours of operation; increased days of operation; space availability; new products accessibility; merging with other business; new stores	*n* = 76 (23%)
Policy	Laws and regulations and public safety: decriminalization; developing marijuana-specific laws; law enforcement; legalization; limited licensed retailers; lowering taxation; people’s perception on cannabis regulation; age restriction; use in the public place; car safety; road safety	*n* = 71 (22%)
Education/information	Advertisement of the store and service; advocacy for cannabis use; awareness on the effects of cannabis; overdose; use (medical and non-medical); different strains; economic impact of the industry; comparison between alcoholism and cannabis use; health effects; inform the consumers about new information; providing access to documents related to cannabis use; research findings; use of educators	*n* = 49 (15%)
Uses of cannabis	Regular use; frequent use; medical use; non-medical use	*n* = 48 (15%)
Cannabis product	Packaging—Inadequate packaging; use of recycled materials for packaging (*n* = 2) Price—compassionate pricing program; high price; need of price reduction (*n* = 4) Quality—potency; percentage of THC and CBD; aroma; taste; visual appeal (*n* = 18) Source—licensed producers; retail; legal source; illegal; homegrown (*n* = 13) Type of product—edibles; cannabis concentrate; hybrid strain (*n* = 9)	*n* = 46 (14%)
Social (gender + stigma)	Gender—women in the industry Stigma—lack of recognition; cannabis in sport; lack of education; racial justice; misconception; promoting the end of stigma	*n* = 20 (6%)
COVID-19 impacts	Reduced access to stores; closure of stores; community service; non-touch delivery; employee health concern; limited payment option; reduced store capacity; reduced store hours; struggle of small business; stocking up	*n* = 19 (6%)
	Total =	329

#### Access to cannabis

The greatest number of tweets by licensed Canadian retailers focused on access to cannabis products. In these tweets, retailers primarily discussed the increased accessibility to the consumers (e.g., increased hours and days of operation and larger space availability):

“We announce, with pride, the grand opening of our flagship retail store. This large space mixes a retail shop with an immersive space where cannabis, community, and culture come to life.”

Access was also increased through online ordering and payment systems (e.g., mobile apps) and easy deliveries (e.g., home delivery, free delivery).

“Someone spoke about scaling the unscalable which led us to offer free delivery. Next month we are raising the standard by ensuring that our website has up-to-date information on package dates, the percentage of THC based on what we have.”

Cannabis licensed retailers also talked about continuous access to the products, easy accessibility to stores (e.g., store close to bus/subway stations, located in easily accessible neighborhoods), and access to different types of products (e.g., edibles).

Limited access to the stores due to security issues, limited ability to provide online access to consumers, and delay in delivery due to less supply or increased demands of products were also discussed among licensed retailers.

“All stores in this area will be closed due to new incidents in the area as a precaution to protect our employees and our customers. Sorry for the inconvenience.”

These retailers also tweeted about merging with other businesses and starting new stores as ways of increasing accessibility to cannabis buyers.

#### Cannabis policy

The second-highest number of tweets was about policy. There appeared to be an overall satisfaction with the legalization of recreational cannabis use.

“Fantastic! The Senate just passed the third reading of the Cannabis Act in Canada. Historic times. #BillC45.”

Despite the satisfaction, there were tweets related to cannabis laws and their enforcement, lowering taxation on medical cannabis, people’s perception of cannabis regulations, and decriminalization of cannabis product possession.

“It is wrong to tax medical cannabis. It is outrageous. It is the only medicine that’s taxed.”

“Amazing news that a legislation will be put forward to seek blanket pardons for personal possession of cannabis offences. #RightingHistoryWrongs.”

#### Cannabis education/information

Licensed retailers extensively tweeted on topics related to cannabis education/information. They discussed information including various available strains of cannabis, different uses of products, and health effects.

“We are aware of THC and CBD, but not dozens of other cannabinoids and compounds in a cannabis flower. The impact of the interaction of those in the body is called Entourage Effect.”

“Learn how THC tolerance develops, and how your tolerance level recovers soon after you take a break.”

Information was also focused on the differential effects of alcohol and cannabis products, advocacy for cannabis, and the availability of relevant information. A few of the tweets were targeted to inform people about the economic impacts of the industry on the economy of the country.

#### Uses of cannabis

Cannabis retailers discussed different reasons for consuming cannabis (e.g., medical or non-medical use) and various types of users (e.g., regular users, frequent users). Tweets like “How Cannabis and CBD Oil Helped Relieve My Daughter’s Chronic Illness Symptoms!” describe cannabis retailers’ views on its use.

### Unlicensed cannabis retailers

Tweets from unlicensed retailers were more focused on education and information about cannabis (37%), use of cannabis (25%), and cannabis products (20%), including tweets about the quality, types, and sources of cannabis products (Table 3). Only 1% of the tweets were related to COVID-19 impact on cannabis use. We described the three most frequent themes and a unique finding on “free giveaways” by unlicensed retailers.

**Table 3:** Themes and nodes in unlicensed cannabis retailers

Theme	Nodes	Number and percentage of tweets
Access	Easy access to store; limited access; online access; barriers to online access; home delivery; hours of operation; space availability; new products accessibility	*n* = 15 (4%)
Policy	Decriminalization; developing marijuana-specific laws; law enforcement; legalization; limited licensed retailers; age restriction; use in the public place; overdose	*n* = 38 (10%)
Education/information	Addiction; advertisement of the store and service; information on cannabis plant; harvesting; use (medical and non-medical); and different strains; economic impact of the industry; comparison between alcoholism and cannabis use; health effects; historical use of cannabis; inform the consumers about a lack of information; creating online community; providing access to documents related to cannabis use; promoting weed use; research findings; use of educators; use of vaping device	*n* = 140 (37%)
Uses of cannabis	Edibles; frequent use; medical use; non-medical use; recreational use	*n* = 95 (25%)
Cannabis products	Packing—glorious packaging (*n* = 2) Quality—potency; percentage of THC and CBD; aroma; taste; visual appeal (*n* = 33) Source—illegal; homegrown (*n* = 18) Type of product—edibles; cannabis concentrate; hybrid strain (*n* = 21)	*n* = 74 (205%)
Social (gender and stigma)	About addiction; cannabis use; criminalization; health effects; misconception; promoting the end of stigma; publicly used by baseball players	*n* = 11 (3%)
COVID-19 impacts	Reduced access to stores; increased sale	*n* = 3 (1%)
	Total =	376

#### Education/information

Under the theme of information and education, tweets were about different types of cannabis plants, harvesting procedures, different strains, promoting weed uses, advertisement of the store and services, cannabis contribution to the economy, the difference between alcoholism and cannabis consumption, cannabis addiction, and cannabis effects on health (Table 3).

“Scientists recently identified two new compounds in cannabis. One of which, THCP, may be a game changer in our knowledge of the plant.”

“One way to check up the health of plants is pruning where the gardener looks closely at the plants.”

A considerable number of tweets talked about the historical use of cannabis, the comparison between alcoholism and cannabis use, cannabis addiction, and its effects on health.

A few tweets highlighted a lack of information on cannabis use, access to documents related to promoting weed use, creating an online community of consumers, and research findings on the positive effects of cannabis on health. Unlicensed retailers also discussed the use of educators to inform people about cannabis harvesting and the use of vaping devices.

#### Use of cannabis products

Unlicensed retailers used tweets to inform consumers about different types of cannabis uses such as medical and non-medical use.

“Cannabis may be called many names, and I call it medicine.”

“Smoking the flower reveals you to yourself.”

They also tweeted about using vaping devices for cannabis consumption. A considerable number of tweets were on the use of specific types of cannabis products, such as edibles, concentrated cannabis, and hybrid strains. Many of the products were from homegrown sources.

#### Cannabis products

A large number of tweets by unlicensed retailers discussed information related to packaging, quality of cannabis products, and types of products. Quality by unlicensed retailers often referred to potency, aroma, taste, and visual appeal.

“These colors are fantastic!”

“The plants look beautiful at the end of the sixth week. We are growing what is known as Cherry Bomb/Cherry OG cross.”

To promote the products, unlicensed retailers relied on the perception of quality and glamorous packaging.

#### Free giveaways by the unlicensed retailers

One of the unique findings while conducting the content analysis of tweets by unlicensed retailers was that a large number of most-liked and favorited tweets were about free giveaways (Table 4).

**Table 4:** Giveaways from the unlicensed retailers

Category	Types of product	Percentage
Cannabis product	Canned organic cannabis; cannabis concentrate; cannabinoid crumble; edible cannabis; CBD oil for pain relief; pharmaceutical-grade cannabis; medicated edibles; hybrid cannabis	*n* = 135 (57%)
Personal care product	Cannabis personal care products; CBD personal care products	*n* = 4 (2%)
Vaping device	Vape pen; vape cartridges	*n* = 25 (10.5%)
Others	Advent calendar; spice grinder; propane hash oil	*n* = 6 (2.5%)
Not declared	Asked to contact the adviser	*n* = 68 (28%)

Those tweets were pulled out to allow for tweets with other themes to be included in the analysis. Our study found that free giveaways included cannabis products such as canned organic cannabis, concentrates, CBD oil for pain relief, and cannabinoid crumble among other things, cannabis personal care products, vaping devices, and other stuff.

“It’s CannabisMoHappyMonday Giveaway! Our biggest prize this week will be:

1 x Gone Girl - Medi Tab – Rasp Blueberry

1 x Gone Girl - Medi Wall - Chocolate Peanut

ReTweet+Like to #WIN.

Browse our store at … [link].”

A large number of tweets did not identify the free giveaway, but asked the Twitter users to “contact the adviser.”

### General tweets gathered using keywords

Tweets collected through keywords were mainly about information/education (31%), access (19%), and use of cannabis (19%). Product-related tweets (18%) were about the source and the quality of the product, packaging, and price. Policy-relevant tweets spoke of public safety, legalization, small-business friendly regulations, and lowering taxation (Table 5).

**Table 5:** Themes and nodes in keyword search

Theme	Nodes	Number and percentage of tweets
Access	Store close to the community; stores in other community facilities; diversifying store locations; continuous access; easy access to store; limited access; online access; finding a store; user-friendly space; developing mobile apps; barriers to online access; delay in delivery; home delivery; same-day delivery; increased hours of operation; increased days of operation; space availability; local products; local stores; loss of local business; loss of products; local people friendly marketing model; new products accessibility	*n* = 62 (19%)
Policy	Decriminalization; small business-friendly laws; law enforcement; legalization; limited licensed retailers; lowering taxation; people’s perception on cannabis regulation; age restriction; use in the public place; road safety	*n* = 32 (10%)
Education/information	Advertisement of the products; advocacy for cannabis use; economic contribution; contribution to society; facts on cannabis; harvesting; use of educators; use of media; awareness on the effects of cannabis; overdose; use (medical and non-medical); and different strains; economic impact of the industry; health effects; new information; cannabis history; providing access to documents related to cannabis use; research findings; industry growth and expectations; community support; social media use	*n* = 102 (31%)
Uses of cannabis	Frequent use; medical use; non-medical use; recreational; illicit user	*n* = 62 (19%)
Cannabis products packaging	Packaging—inadequate packaging; over packaging; inadequate labeling (*n* = 5) Price—high price; need of price reduction (*n* = 5) Quality—potency; percentage of THC and CBD; aroma; taste; visual appeal (*n* = 24) Source—licensed producers; legal source; illegal; black market; homegrown; government sources; retail market (*n* = 22) Types—edibles (*n* = 1)	*n* = 59 (18%)
Social (gender and stigma)	Mental health; lack of education; misconception; promoting the end of stigma; community involvement; women targeted product	*n* = 6 (2%)
COVID-19 impacts	Reduced access to stores; closure of stores; losing retailers; non-touch delivery; reduced access to products; reduced store hours; struggle of small business	*n* = 7 (2%)
	Total **=**	330

#### Education/information

With regard to education and information, a majority of tweets gathered through keywords were about advocacy for cannabis use, providing access to documents related to cannabis, facts on cannabis (medical and non-medical), community support, and its contribution to society and economy (Table 5).

“Seven Common Myths About Cannabis. #education #misconceptions #myths”

Some of the accounts advertised different cannabis products (including different strains), harvesting techniques, and the role of media. Tweeters used this social platform to increase awareness of the effects of cannabis, overdose, and health effects. There was one tweet on the contribution of legal cannabis to the economy.

“The Statistic Canada’s Figures today show legal cannabis industry has grown by 11% in May from a year earlier. Similarly, the unlicensed market’s contribution to Canada’s GDP has fallen by about 13% in that same time”.

#### Access to cannabis products

Issues of access to cannabis were discussed frequently such as store close to the community, diversifying store locations, continuous access, easy access to a store, online access, ways to find a store, user-friendly spaces, developing mobile apps to access cannabis, barriers to online access, delay in delivery, home delivery, same-day delivery, increase hours and days of operation, local products, local stores, friendly marketing, and new products accessibility.

“Re-zoning of cannabis retail continues in the District of North Van. Three spanning different regions of the municipality has been approved.”

Some of the tweets mentioned the barriers to online access, delay in delivery, and too many stores in a particular community.

“While Cannabis stores provide a service in our communities, the clustering of them in one area undermines opportunities for more diverse retail.”

#### Uses of cannabis

On uses of cannabis, the most common nodes were on medical use, non-medical use, and frequency of use. A few tweets mentioned that cannabis was a medicine and referred to its impact on dealing with a disability.

“I say cannabis is a medicine because of this. Look how a few puffs help this man cope with a debility.”

#### Cannabis products

For cannabis quality, the tweets spoke of cannabis products such as potency, percentage of THC and CBD, aroma, taste, and visual appeal of the products. Regarding the sources of the cannabis, tweets were about licensed products and unlicensed producers in the illegal market and homegrown products versus corporate production. A few tweets raised the issue of inadequate packaging and labeling.

## Discussion

This study conducted sentiment and content analyses of Twitter data about cannabis in Canada. This analysis of cannabis-related tweets found commonalities and differences in tweets’ sentiment and content among licensed retailers, unlicensed retailers, and general tweets using keywords. All of those groups expressed their views on a diverse group of topics including education and information on cannabis, accessibility, cannabis-related policies including public safety, uses of cannabis, cannabis products including price, sources, and types, packaging, and social issues like gender and stigma.

This study found positive sentiments and positive emotion rates on cannabis in Canada, which may indicate the effects of the legalization of cannabis, as people became more comfortable sharing opinions and emotions on online social platforms. However, other studies have also shown predominantly positive attitudes toward cannabis in the United States (Cavazos-Rehg et al. 2015; Lamy et al. 2016).

Licensed retailers tweeted mainly on access to cannabis products, cannabis-related policies, education and information, and uses of cannabis products. Providing information on cannabis use, harvesting, quality of cannabis products, and advertising were the focus of the unlicensed retailers’ tweets. The similarities in the themes might be because both licensed and unlicensed retailers focused their discussions on the users’ common interests. The differences between them may be explained because all tweets by licensed retailers were posted after the legalization and legality of the licensed retailers. However, we did not notice any dramatic change in messaging by unlicensed retailers since legalization. Besides, tweets collected through keywords were mainly about information/education, access, and use of cannabis (medical and non-medical).

Though the education/information theme was commonly discussed by both licensed and unlicensed retailers, it constituted the highest proportion of tweets for the latter group. Licensed retailers mainly provided information on the store and service, use of cannabis for medical purposes, awareness of the effects of cannabis, health effects such as overdose, the economic impact of the industry, new information on cannabis, access to documents related to cannabis, and research findings on cannabis. On the other hand, unlicensed retailers discussed different types of cannabis plants and strains, harvesting, the industry’s economic impact, comparison between alcoholism and cannabis, historical use of cannabis, and educators’ use for informing the general public about cannabis and vaping. General tweets focused on local products, local stores, and local people-friendly marketing models. This might imply that, after the legalization of non-medical use of cannabis in Canada, cannabis consumption may be becoming more popular and they are trying to find different options for cannabis accessibility.

Previous Twitter content analysis of cannabis revealed themes such as users’ intent to use, health benefits, sex/romance, and legalization (Cavazos-Rehg et al. 2015). This content analysis revealed some similar themes found in previous studies, such as policy (i.e., legalization), use (medical and non-medical) (West et al. 2012), sources (Allem et al. 2020; Cavazos-Rehg et al. 2014), and types (e.g., cannabis concentrate, hemp) (Darren et al. 2020). However, our study also presented new themes like information/education, effects of the COVID-19 pandemic on cannabis use and industry, and public safety. Moreover, our analysis did not identify some themes that other authors found, such as tobacco use, romance, and friendship (Cavazos-Rehg et al. 2015). This could be because of our Tweet search or focus on tweets from three groups, namely tweets from “Cannabis Licensed Retailers” and “Cannabis Unlicensed Retailers,” and tweets gathered through specific keywords. Whereas some previous studies focused on tweets from adolescents (Cabrera-Nguyen et al. 2016; Cavazos-Rehg et al. 2014; Cavazos-Rehg et al. 2015; Krauss et al. 2016), our keyword search gathered Tweets from the general population.

Tweets in this study also showed that in-store shopping reduced significantly during the COVID-19 pandemic, but the overall sales increased, most probably through online shopping. The current tweets also revealed the growing popularity of cannabis consumption that enhanced discussions on the accessibility to cannabis, including easy access to a store, limited access, online access, barriers to online access, home delivery, hours of operation, space availability, and new products accessibility. COVID-19 might be another reason for raising the issues on cannabis use. In a study on populations in the Netherlands, Laar et al. (2020) showed that 41.3% of participants increased their cannabis use after lockdown due to COVID-19. Although it has not been proven, some suggested that cannabis might prevent COVID-19 (Pascual Pastor et al. 2020; Hill 2020), which might have increased the number of consumers. Their concerns might be another reason for a higher number of tweets on cannabis use.

As 3 years have passed since cannabis legalization in Canada, the social media sphere of cannabis is perhaps the least-watched and regulated in Canada. Although advertising cannabis is banned, unlicensed retailers are using Twitter to promote their products by giving away free cannabis and cannabis products to Twitter users. As a most frequented media space by the general population and youth and young adults, social media offers an opportunity for policy makers to inform, educate, and interact with the public on issues related to cannabis. The two-way interaction on social media can be untapped to identify important themes for the public on cannabis to revise policies as we move to better regulate cannabis in the country.

Our study was limited to analyzing tweets from specific accounts and tweets containing specific words or terms, English language contents, and those from Canada. We also did not distinguish between bot and non-bot accounts, which may have influenced the sentimental analysis. Moreover, geotagged Twitter data is a subset of general Twitter data that may not accurately represent the general population. Therefore, the social media data used in this study were from a non-representative subset of the Canadian population. Tweets offer short snapshots of issues, do not offer in-depth insights, can set the agenda and themes on cannabis, but would not offer a contextual explanation.

## Conclusion

Canadian public sentiment on cannabis expressed through Twitter data was positive, and major themes included information and education on cannabis; use of and access to cannabis products; policies around cannabis, quality, source, and types of cannabis products; social stigma; and COVID-19 impact on cannabis. This study combined the power of big data collection and analysis with manual coding to extract rich content from large data using social media communications on issues related to cannabis. The content analysis highlighted two potential themes that deserve further investigation: using social media for cannabis product promotion by retailers and using social media platforms by public health practitioners to inform and engage cannabis users about evidence-based information on cannabis. Knowledge generated from this study may inform Canadian cannabis policy revisions as more than three years have passed since the legalization of cannabis.

## Data Availability

The data can be made available upon request.

## Appendix 1

Table 6

**Table 6:** Twitter handles of licensed and unlicensed retailers in Canada

No	Licensed retailers	Twitter handle	Followers
1	Aurora Cannabis - W Edmonton Mall	@Aurora_MMJ	42,500
2	Tweed	@TweedInc	28,700
3	NSLC	@theNSLC	15,700
4	Tokyo Smoke	@tokyo_smoke	8773
5	Delta 9 Cannabis Store	@Delta9BioTech	7935
6	The Original FARM	@FarmCannabis	7388
7	SQDC (Société québécoise du cannabis)	@La_SQDC	4646
8	Hotbox Shop Kensington Market	@hotboxcafe	3674
9	Spiritleaf	@SpiritleafCA	2474
10	Friendly Stranger	@friendlystrnger	2454
11	Meta Cannabis Supply Co.	@themetaco	2289
12	Fire & Flower	@fireandflowerco	2286
13	Choom	@choombrand	1487
14	Calyx + Trichomes	@CalyxTrichomes	1404
15	NewLeaf Cannabis	@newleafcannabis	1299
16	Pineapple Express	@PXDelivery	1246
17	Summit Cannabis Co	@SummitCanCo	1221
18	Village Bloomery	@villagebloomery	1135
19	Star Buds	@StarbudsCanada	1081
20	MariJane Depot	@MarijaneSociety	21,500
21	Green Society	@greensocietyca	5675
22	DispensaryGTA	**@**dispensarygta	5536
23	BMWO	**@**BuyMyWeedOnline	4532
24	BudMail	**@**therealbudmail	2564
25	Cannabismo	**@**officialcbismo	2375
26	PlatinumHerbalCare	**@**platinum_herbal	2028
27	Hillside Pharms	**@**HillsidePharms	1522
28	Cannabisy	**@**cannabisyinfo	1437
29	Top Leaf(legal)	**@**TopLeafCan	1086
30	HerbStoreBC	**@**herb_bc	1020

## Appendix 2

Table 7

**Table 7:** Keywords related to Cannabis

Cannabis	cannabislife	hemp	smokeweedeveryday
cannabisbusiness	cannabislifestyle	hempnews	Spliff
cannabisbutter	cannabismedicinal	Herb	staylifted
CannabisCanada	cannabisnews	highlife	stoner
cannabisclub	cannabisoil	iamcannabis	stonerbabe
cannabiscommunity	cannabisphotography	Indica	stonerdad
cannabiscu	cannabisretailing	Kush	stonerfam
cannabiscultivation	cannabissociety	Marijuana	stonergirl
cannabisculture	cannabutter	marijuananews	stonergirls
cannabiscures	cannafam	MARYJANE	stonerthoughts
cannabisdaily	cannahoney	medicalcannabis	Swishers
cannabisdaily	cannibiscures	medicalmarijuana	Terpines
cannabisenthusiast	ediblecannabis	Mj	THC
cannabisgrow	flowerpower	mmemberville	wakeandbake
cannabisgrower	Ganja	Mmj	Weed
cannabisheals	gethigh	mmot	weedbutter
cannabisindustry	growcannabis	pothead	weedcommunity
cannabisindustry	growweed	potstocks	weedlife
cannabisinvesting	growyourown	Reefer	weedlovers
cannabisisessential	Hashish	Sativa	weedstagram

## Appendix 3

Table 8

**Table 8:** Twitter data dictionary

Field	Type	Description
text	String	The actual UTF-8 text of the tweet
id	String	The string representation of the unique identifier for this Tweet.
retweet_count	Int	Number of times this Tweet has been retweeted
favorite_count	Int	Nullable. Indicates approximately how many times this Tweet has been liked by Twitter users.
created_at	String	UTC time when this Tweet was created
hashtags	String	Hashtags
user_id	String	The string representation of the unique identifier for this user
user_screen_name	String	user_screen_name
user_location	String	user_location
user_followers_count	int	Number of the user’s followers
user_friends_count	int	Number of the user’s friends
user_created_at	String	UTC time when this user was created
user_geo_enabled	boolean	user_geo_enabled
coordinates	coordinates	Nullable. Represents the geographic location of this Tweet as reported by the user or client application
place	place	Nullable When present, indicates that the tweet is associated (but not necessarily originating from) a Place
in_reply_to_status_id	String	Nullable. If the represented Tweet is a reply, this field will contain the string representation of the original Tweet’s ID
in_reply_to_user_id	String	Nullable. If the represented Tweet is a reply, this field will contain the string representation of the original Tweet’s author ID. This will not necessarily always be the user directly mentioned in the Tweet.
quoted_status	Tweet	This field only surfaces when the Tweet is a quote Tweet. This attribute contains the Tweet object of the original Tweet that was quoted.
retweeted_status	Tweet	This attribute contains a representation of the original Tweet that was retweeted. Note that retweets of retweets do not show representations of the intermediary retweet, but only the original Tweet.
reply_count	int	Number of times this Tweet has been replied to

## Abbreviations

API: Application Programming Interface
CBD: Cannabidiol
THC: Tetrahydrocannabinol
